# Ultrasound findings in pregnant women with uncomplicated vivax malaria in the Brazilian Amazon: a cohort study

**DOI:** 10.1186/s12936-015-0627-1

**Published:** 2015-04-08

**Authors:** Marianna F Brock, Angélica E Miranda, Camila Bôtto-Menezes, Jorge RT Leão, Flor E Martinez-Espinosa

**Affiliations:** Fundação de Medicina Tropical Dr. Heitor Vieira Dourado, Av Pedro Teixeira 25, 69040-000 Manaus, Amazonas Brasil; Universidade do Estado do Amazonas, Av Castelo Branco 1777, Manaus, Amazonas Brasil; Universidade Federal do Espírito Santo, Vitória, Espirito Santo Brasil; Instituto Leônidas e Maria Deane, FIOCRUZ Amazonas, R Terezina 476, 69057070 Manaus, Amazonas Brasil

**Keywords:** Malaria, Vivax, Ultrasonography, Pregnancy, Amazon, Placental thickness

## Abstract

**Background:**

During pregnancy, *Plasmodium falciparum*-induced malaria can cause placental lesions and intrauterine growth restriction (IUGR). There are few published studies on *Plasmodium vivax*-induced malaria in pregnancy. Ultrasound is an efficient method for evaluating foetal biometry and placenta. The present study aimed to investigate the occurrence of increased placental thickness, foetal biometry and the amniotic fluid via ultrasound in a cohort of pregnant women with vivax malaria in Manaus, Amazonas, Brazil.

**Methods:**

A cohort study was conducted among 118 pregnant women with vivax malaria and 191 pregnant women without malaria. Foetal biometry, placental thicknesses and the amniotic fluid were evaluated via ultrasound. Biometric data were distributed by the trimester in which the infection occurred and converted to Z scores. The results were compared between the groups.

**Results:**

Among pregnant women from the cohort, increased placental thickness was observed in ten women with malaria (8.5 *vs* 0%; p <0.001). The Z scores of biometric parameters were not statistically significant when comparing the groups or according to the time of infection. In ultrasound results of the 118 pregnant women with malaria, seven (6%) showed low foetal weight, two (1.7%) showed oligohydramnios and one (0.85%) showed foetal malformation. There was no significant difference when these variables were compared to those of the control group.

**Conclusions:**

The placental thickness changes were significant but caused no foetal repercussions at birth. The ultrasound findings except placental thickness were similar in both groups, possibly because this is a low-endemic area and the pregnant women in the study were followed up in an active detection system that allowed early diagnosis and treatment of new malaria episodes.

**Electronic supplementary material:**

The online version of this article (doi:10.1186/s12936-015-0627-1) contains supplementary material, which is available to authorized users.

## Background

Malaria is among the most important public health issues worldwide [1]. In Latin America, it is estimated four in every 100 pregnancies are affected by malaria, and there is a high incidence in Brazil [2]. In 2011, 99.7% of malarial transmission was concentrated within the Amazonian region [1-4], where malaria transmission is considered low and unstable and the most prevalent parasite species is *Plasmodium vivax* [1,5,6].

Pregnant women are more susceptible than the general population to *Plasmodium sp.* infections [7], and malaria can convert normal pregnancies into pathological pregnancies [8-11]. During pregnancy, *Plasmodium falciparum*-induced malaria can cause placental lesions and changes in foetal oxygenation that lead to neonatal impairments, such as intrauterine growth restriction (IUGR), decreased amniotic fluid, prematurity and brain injuries caused by hypoxia and intrauterine death [12-14]. Studies in Thailand, India and Bolivia showed that compared to non-infected women, *P. vivax*-infected pregnant women are more often anaemic and have newborns with lower birth weights [3,15-17].

Ultrasound is a useful tool for detecting IUGR and determining the gestational age by foetal biometry [18]. Placental ultrasound can assess the placental thickness, texture and maturity level, and thus contributes to diagnoses of placental insufficiency [19,20]. Placental thickness has been associated with various maternal and foetal conditions, including infections (toxoplasmosis, rubella, cytomegalovirus, and other infections), diabetes, anaemia, and hydrops, while decreased placental thickness has been associated with pre-eclampsia, thyroid disease, placenta insufficiency and IUGR [21-23].

In cases of placental insufficiency and chronic hypoxia, the foetus adapts to the new situation by protecting its important organs. This mechanism leads to decreased diuresis and secretion of pulmonary fluids and subsequently leads to oligohydramnios, which is usually detected via ultrasound [24]

There is a lack of studies on ultrasound evaluations of foetal growth, amniotic fluid and placentas in pregnant women with vivax malaria. The present study aimed to investigate the occurrence of changes in placenta, foetal growth and amniotic fluid volumes by ultrasound in a cohort of pregnant women with vivax malaria in Manaus, Amazonas, Brazil.

## Methods

A prospective cohort study of pregnant women with and without vivax malaria, all of whom had gold standard first trimester ultrasound dating scans (CRL ≤14 wks), were enrolled from January 2006 to July 2007 in the city of Manaus at the Dr Heitor Vieira Dourado Tropical Medicine Foundation (Fundação de Medicina Tropical Dr Heitor Vieira Dourado; FMT-HVD). This institution is also a primary care centre, diagnosing and treating patients with malaria even if they have no clinical complications. The unexposed group was selected from another close health unit devoted to antenatal care, attending patients with the same epidemiological profile in the same period, matched by maternal age and gestational age at inclusion.

The unexposed group was recruited in another clinic because the FMT-HVD is a reference hospital for infectious disease and infections in pregnancy were an excluding factor in these study (they can lead to IUGR and other diseases). The study design planned to include one unexposed woman for each exposed pregnant woman. Most of the population from Manaus is of mixed races, therefore, ethnic group was not specified. Routine blood examinations were performed in all of them. The same physician performed the ultrasound in both groups.

### Malaria diagnosis

The microscopic diagnosis was performed by thick blood smear in all women. Maternal parasite load was estimated using a semi-quantitative method as recommended by the Brazilian Ministry of Health: 200–300 parasites per cu mm; 301–500 parasites/cu mm; 501–10,000 parasites/cu mm; 10,001-100,000 parasites/cu mm and 100,000 or more parasites/cu mm.

Parasite densities with differential counting of asexual stages were estimated by experienced microscopists, by counting the number of parasites in 200 leukocytes in high magnification fields, and the number of leukocytes/cu mm.

The thick blood smear was performed at the moment of recruitment and monthly when the patient returned to antenatal evaluation.

### Study procedures

The patients recruited for the study were interviewed and responded to a questionnaire regarding clinical and demographic data and were subsequently referred to ultrasound evaluation. The ultrasound examinations were performed at the time of the malaria diagnosis and at 30-day intervals until the delivery date. The examinations were performed with the GE Voluson Expert and GE P6 devices (GE Healthcare, Little Chalfont, Buckinghamshire, UK) by a single examiner who was certified in ultrasound examination by the Brazilian College of Radiology (Colégio Brasileiro de Radiologia) and the Brazilian Federation of Gynecology and Obstetrics (Federação Brasileira de Ginecologia e Obstetrícia) and in Foetal Medicine by the Brazilian Federation of Gynecology and Obstetrics.

### Ultrasound examination

The patients were examined while in the supine position for bi-dimensional evaluations. They were instructed to relax the abdominal muscles and not to position the arms under the head or cross the legs in order to prevent muscle contracture. The gestational age was estimated from the crown-rump length (CRL) during first trimester ultrasounds, according to the technique reported by Grisolia *et al.* [25]. During the second and third trimesters, foetal biometry was based on the biparietal diameter (BPD), head circumference (HC), abdominal circumference (AC), and femur length (FL) The BPD and HC were measured according to Campbell and Thoms [26]; the AC was measured according to Campbell and Wilkin [27]; and, the FL was measured according to Kurmanavicius [28]. From 18 to 24 weeks, a foetal morphological study was performed and, in addition to measuring the biometry based on the long bone evaluation as described above, the tibia, fibula, radius, ulna, foot, and cerebellum were also measured to evaluate the foetal morphology. The biometric parameters based on the BPD, HC, AC, FL, and weight were measured according to the Hadlock table [29]. Three measurements were obtained for each parameter, and the means of the three values were calculated. The professional that performed the ultrasound knew to which group each pregnant woman belonged.

In an attempt to eliminate problems caused by variations in the measurements according to the gestational age, the Z score was used to compare the ultrasound results between the two groups. When the score was two standard deviations or more above or below the mean, it was considered altered.

The Z scores were evaluated for all parameters according to the following formula: Z = (X-M(GA))/SD(GA), where X is the measured value (BPD, FL, HC and AC), M(GA) is the mean of the approximate value for the evaluated gestational age according to the normality, and SD(GA) is the standard deviation of the mean for the gestational age according to the standard curve.

The amniotic fluid was evaluated according to the amniotic fluid index (AFI) proposed by Phelan *et al*. [30]. The placental thickness and location were evaluated at a perpendicular plan to placental axis at the point of insertion of the umbilical cord, according to Perroti [31]. The results of placental thickness were presented in millimetres. The ultrasound results were analysed with perinatal data and data from the unexposed group. It is important to take into account that after >24 weeks ultrasound scans are prone to high rates of error on the measurements, but by using a control group this limitation is minimized. In both groups the following perinatal data were evaluated: gestational age at birth, birth type, foetal weight at birth, presence of malformations, and maternal parity.

In the group of pregnant women with malaria, the treatment comprised the use of chloroquine (1,500 mg for three days and then weekly prophylaxis with 300 mg of chloroquine for 12 weeks), according to the recommendation of the Brazilian Ministry of Health [32]. In patients diagnosed with anaemia, ferrous sulphate (200 mg, three times/day) and folic acid (5 mg/day) were also prescribed according to the recommendation of the Brazilian Ministry of Health [33].

In both groups, obstetric complications were treated at the local maternity hospitals in the region, according to proximity to the patient’s house; the births also occurred at these hospitals. Approximately one month after delivery, the patients came back to another medical evaluation, and newborn data such as body weight, Apgar scores and the presence of complications were collected from the newborn cards that were filled out at the maternity hospitals.

### Definitions of variables

- Vivax malaria: presence of *P. vivax* on the microscopic thick film examination;
- Maternal anaemia: haemoglobin lower than 11 mg/dl;
- Maternal weight: measured upon enrolment and at each antenatal consultation until childbirth. Mother’s weight prior to pregnancy was based on antenatal dates. Weight gain was defined as the difference in maternal weight between the first and last weight measured at antenatal consultation;
- Low ultrasound weight: weight less than the tenth percentile for the gestational age;
- Low weight at birth: weight at birth less than the tenth percentile for the gestational age. (Newborns birth weight was measured immediately after birth as Ministry of Health recommendation) [34];
- Pre-term babies: newborns with gestational age less than 37 weeks according to the first trimester ultrasound;
- IUGR: foetuses at term with low weight (less than the tenth percentile according to gestational age proposed by Olsen [35];
- Abortion: birth that occurred before 20 weeks of age;
- Congenital abnormality: an abnormality that was present on the ultrasound and diagnosed by the trained neonatal doctor during the newborn examination;
- Increased placental thickness: placenta above the 90th percentile for the gestational age [36].

### Inclusion and exclusion criteria

Patients with pregnancy diagnoses that were confirmed by a quantitative beta human chorionic gonadotropin test or an abdominal or transvaginal obstetric ultrasound during the first trimester, and who agreed to participate by providing informed, signed consent were included in the analysis.

Patients with non-vivax malaria, a previous diagnosis of maternal disease or any other factors that could affect the foetal growth or morphology of the foetus, such as hypertension, auto-immune disease, other infection, multiple pregnancy and diabetes (diagnosed by fasting blood glucose and/or oral glucose tolerance test), were excluded from the study. Patients whose gestational age could not be evaluated correctly because they did not undergo a first trimester ultrasound and patients who did not attend the follow-up examinations were also excluded from the analysis.

### Statistical analysis

The results were tabulated with Excel (Microsoft Corporation, Redmond, WA, USA) and analysed with Epi-Info for Windows, version 3.5.3 [37] The data were organized and presented in graphs and tables. The variables were descriptively analysed by calculating the absolute and relative frequencies. For the quantitative variables, the analysis was performed by observing the minimum and maximum values and calculating the means. For continuous quantitative data, the Student’s t test was used for normally distributed variables, while the Mann–Whitney U test was used for non-parametric data. Categorical data were compared with the Chi-square test and Fisher’s exact test when appropriate.

### Ethics statement

The patients were informed about their participation in the study, and their consent was obtained through informed consent terms that were signed by the patients or their legal guardians. The present study was approved by the Research Ethics Committee of the Fundação de Medicina Tropical Dr Heitor Vieira Dourado (FMT-HVD) under protocol number 0836–05.

## Results

During the study period, 214 pregnant women with malaria and 289 without malaria were referred for ultrasound examination. Among the group of pregnant women with malaria, three were excluded because of multiple pregnancy, 50 because of concomitant diseases (hypertension, other infections during gestation, triploidy, and diabetes), 20 because of falciparum malaria and 23 due to loss to follow-up (total of patients submitted to analysis was 118). Among the group of pregnant women without malaria, six were excluded because of multiple pregnancy, 53 because of concomitant diseases (hypertension, other infections and diabetes), and 39 due to loss to follow-up (total of patients submitted to analysis was 191).

There were no significant differences between the groups regarding to the mean age, education, parity, haemoglobin levels, and maternal weight gain during pregnancy (Table 1). The mean weight gain during pregnancy was 11.7 kg for malaria group and 13.2 kg among the pregnant group without malaria. In the group of pregnant women with vivax malaria, 81 (68.61%) had the first episode of malaria in lifetime in this pregnancy. Parasite load ranged from 501–10,000 parasites/cu mm. Among the patients with malaria, 13 (11.0%) were included during the first trimester of pregnancy, 73 (61.8%) during the second trimester and 32 (27.2%) during the third trimester (Table 1).

**Table 1:** **Characteristics of pregnant women included in the study population in Amazonas, Brazil**

	**Vivax malaria**	**%**	**Range**	**No malaria**	**%**	**Range**	**P-value**
	**n = 118**			**n = 191**			
	**f**_**i**_			**f**_**i**_			
**Age, years**	24.2 (+/−1.4)	-----	14-39	23.5 (+/−1.9 )	-----	15-39	
**Education**							
1-4 years	40	33.9%		60	31.4%		0.74
5-8 years	45	38.1%		92	48.1%		0.10
9+ years	33	28%		39	20.4%		0.16
**Parity**							
Nuliparous	38	32.2%		78	40.8%		0.16
Secundiparous	28	23.7%		51	26.7%		0.65
Multiparous	52	44.1%		62	32.5%		0.06
**Ga at inclusion**							
1st trimester	13	11.0%		29	15.3%		0.34
2nd trimester	73	61.8%		112	58.6%		0.95
3rd trimester	32	27.2%		50	26.1%		0.92
**Mean gestational age at inclusion (weeks)**							
1st trimester	9.2	-----		9.7	-----		0.15
2nd trimester	19.1	-----		16.9	-----		0.99
3rd trimester	31.1	-----		31.4	-----		0.36
**Hb at inclusion**							
1st trimester	10.4	--	7.2-13.5	11.1	---	7.7-13.8	0.38
2nd trimester	11.0	--	7.4-14.1	11.5	---	8.0-14.3	0.48
3rd trimester	10.9	---	7.6-13.5	11.3	---	8.5-14.1	0.38
**Weigh gain, kg**	11.7		5.3-17.2	13.2		8.4-18.6	0.36
**Placental thickness (mm)**							
2^nd^ trimester	22.5 mm (+/− 3.8 mm)	---	---	17.6 mm (+/− 3.7 mm)	----	----	*0,000*
3^rd^ trimester	34.8 mm (+/− 3.8 mm)	---	----	31.4 mm (+/− 3.9 mm)	-----	-------	*0,000*

Ga, gestational age; Hb, haemoglobin.

f_i_ = simple absolute frequency;

A italicized p-value indicates a statistical difference at a 5% significance level.

### Ultrasound findings

Of the ultrasounds from the 118 pregnant women with malaria, seven (6%) showed low foetal weights, two (1.7%) showed oligohydramnios, and one (0.85%) showed foetal malformation. There was no significant difference when these variables were compared to those of the control group. However, increased placental thickness (above placental thickness average table) was only observed in ten pregnant women with malaria with anterior and posterior placenta (8.5%) (p <0.001; Table 2).

**Table 2:** **Ultrasound findings during pregnancy in women included in the study**

	**Vivax malaria**	**%**	**No malaria**	**%**	**RR**	**P-value**
	**n = 118**		**n = 191**			
	**f**_**i**_		**f**_**i**_			
Low USG weight						
Yes	7	6%	8	4.2%	1.41	0.67
					(0.52-3.80)	
No	111	94%	183	95.8%		
Placental thickness					---	*0.0000*
Yes	10	8.5%	0	0%		
No	108	79.6%	191	100%		
Oligohydramnious						0.68
Yes	2	1.7%	6	3%	0.53	
					(0.11-2.62)	
No	116	98.3%	185	97%		
Foetal malformation						0.70
Yes	1+	0.8%	1*	0.5%	1.61	
					(0.10-25.6)	
No	117	99.2%	190	99.5%		

USG, ultrasonography.

f_i_ = simple absolute frequency; RR = Relative risk; (Confidence level -CI = 95%).

A italicized p-value indicates a statistical difference at a 5% significance level.

+ malformation – harelip *malformation – renal dysplasia.

Among the patients with increased placental thickness, five patients had haemoglobin (Hb) levels above 11 mg/dl and above the malaria group Hb mean (10.9 mg/dl). This group had no cases of IUGR or low birth weight and only one patient had pre-term labour with 34 weeks. None of those patients with increased placental thickness had had oligohydramnios, lateral or fundal placenta. Data from increased placental thickness group are presented in Additional file 1.

Comparing increased placental thickness group with placental normal thickness group, there were no statistical differences between maternal age, parity, mean gestational age, Hb levels at presentation, maternal weight gain, BPD, CC, AC Z-score, birth weight and gestational age at delivery (Table 3).

**Table 3:** **Comparison between pregnant with and without increased placental thickness**

**Variable**	**Normal placental thickness**			**Increased placental thickness** ^**b**^ **n = 10**	**p**
	**Malaria**	**No malaria n = 191**	**Total** ^**a**^		
	**n = 108**		**n = 299**		
Mean age	24.4 (+/− 1.3)	23.5 (sd+/−1.9)	23.8	21.8 (+/−5.3)	0.13
Primigravida	34	78	112	4	--
Gestational age (weeks) (mean)	21.5 (sd+/−1.8)	19.5 ( sd+/− 1.3)	20.2 (sd+/− 1.5)	22.4 (sd+/− 2.5)	0.40
Wt gain (kg)	11.65 (sd+/−1.6)	13.2 (sd +/− 1.9)	12.6 (sd: +/− 1.8)	12.2 (s/d +/−1.25)	0.67
Hb at presentation	10.9 (+/−0.9)	11.3 (+/−1.2)	11.1 (+/− 1.0)	10.5 (+/− 0.7)	0.69
Avg parasite^c^	501-10,000	----	-------	501-10,000	---
N episodes	1.5	-----	--------	1.6	---
Gest age 1st infection (weeks)	21.5	----	------	22.4	0.17
Z score DBP	0.97	0.96	0.96	0.29	0.26
Z score CC	0.02	0.03	0.02	−0.11	0.66
Z score CA	0.22	0.28	0.25	0.17	0.76
Z score F	0.03	0.04	0.03	0.02	0.89
Z score placenta	0.75	−0.26	0.10	1.70	*0.000*
Birth weight (kg)^d^	3.158 (sd+/− 465 )	3.277 (sd+/ -472.5 )	3.234 (sd+/−468 g)	3.383 (sd+/− 469 g)	0.75
GA at delivery (weeks)	38.5 (+/− 2,2)	38.7 (+/− 2,6)	38.6 (+/− 2,4)	38.8 (+/− 2,2)	0.77

^a^All comparisons were made between the groups: increased placental thickness and total of normal placental thickness (malaria normal thickness + no malaria group);

^b^All patients had malaria.

^c^parasites per cu mm;

^d^Birth weight in grams.

A italicized p-value indicates a statistical difference at a 5% significance level.

**Figure 1:**
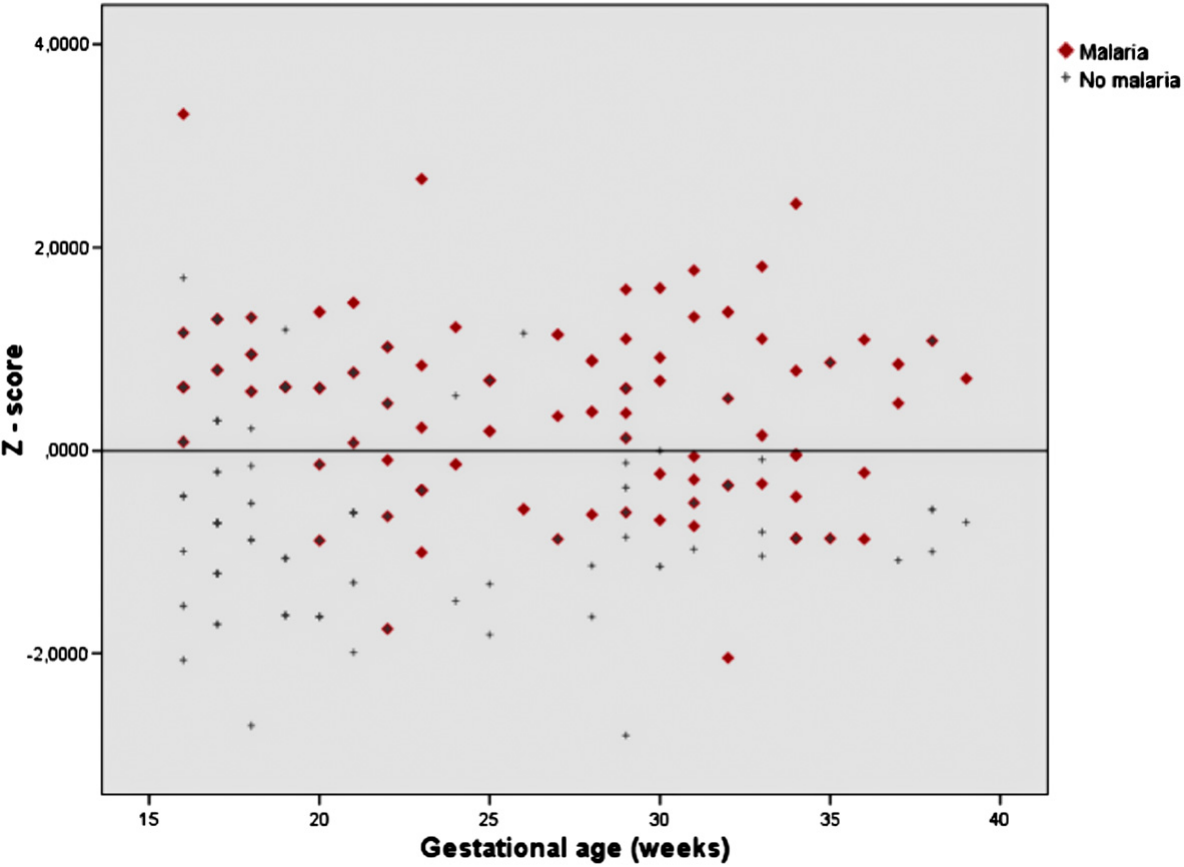
**Relation between placental thickness z-score and gestational age in malaria group (red rhombus) and no malaria group (grey cross).**

Placental analysis revealed a statistically significant increase of thickness in patients with malaria than in the without malaria group (p <0,01) (Figure 1).

### Effects of malaria on BPD, HC, AC, FL, HC/AC ratio, and foetal weight

The ultrasound measurements of foetal growth (BPD, HC, AC, and FL) in the pregnant women with and without malaria were compared to the fifth, 50^th^ and 95^th^ percentiles in the daily use tables, according to the proposal by Hadlock *et al*. [29]. In both groups, the distributions of the evaluated parameters were similar throughout all gestational ages.

The Z scores were compared according to the trimester in which the patients contracted malaria. The first ultrasonography after malaria diagnosis and the last ultrasonography before the birth were compared. When comparing the groups, no significant differences were found in the mean Z scores of the ultrasounds with regard to the trimester of infection p >0.05 in all parameters (Additional file 2). When the Z score data for all trimesters were pooled, there were no significant difference (p >0.05).

**Figure 2:**
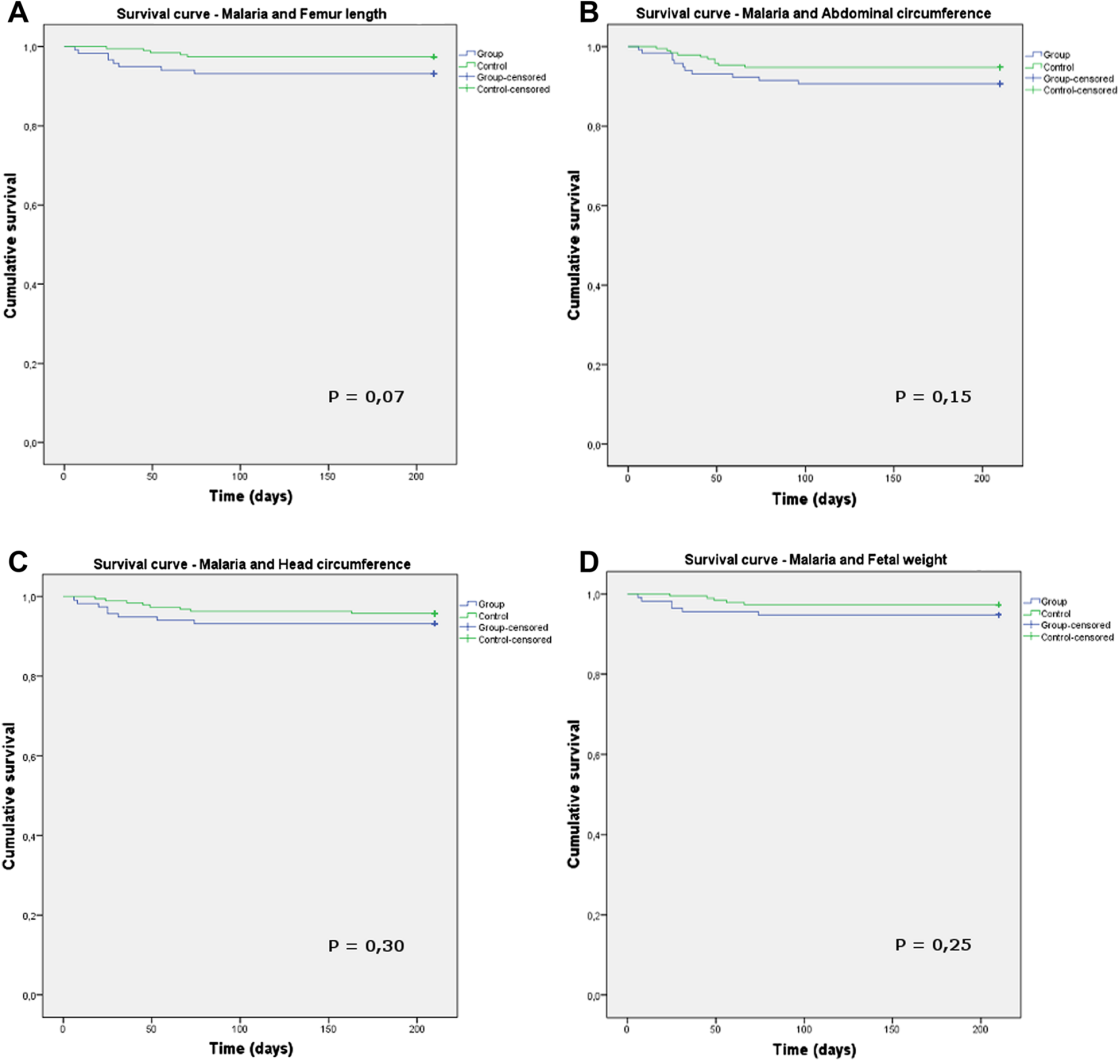
**Survival curves to compare the time of exposure to the occurrence of impairment. A**: malaria *vs* femur length; **B**: malaria vs abdominal circumference; **C**: malaria *vs* head circumference (HC); **D**: malaria *vs* foetal weight. In all graphs: malaria group, blue line; control group, green line. Cumulative survival was defined as the percentage of foetuses that did not have a measurement <2 SD.

To evaluate the effect of the malaria exposure time on foetal changes, survival curves were constructed to analyse the exposure time (in the group with malaria) or the inclusion in the study (group without malaria) with respect to impairment in the foetal weight, BPD, HC, FL, or AC. No significant impairments were found between the groups and the exposure time (Figure 2). An analysis with the same parameters according to the trimester of infection was also performed and did not reveal any significant differences.

### Malaria and perinatal results

Table 4 shows the perinatal results. There were no significant differences between the malaria-exposed and unexposed groups with regard to the perinatal results.

**Table 4:** **Included foetal/newborn data from the study population in Amazonas, Brazil**

	**Vivax malaria**	**%**	**Range**	**Malaria**	**%**	**Range**	**RR**	**p-value**
				**negative**				
	**n = 118**			**n = 191**				
	**f**_**i**_			**f**_**i**_				
Foetal gender								
Male	64	54.3%		102	53.5%		--------	0.97
Female	54	45.7%		89	46.5%		--------	0.64
Birth weight	3,177 g (+/−482.5 g)		1,945-4,715 g	3,277 g (+/− 472.5 g)		590-4,290 g	--------	0.33
LBW yes	7	5.9%		18	9.5%		0.71 (0.37-1.36)	0.37
LBW no	111	94.1%		173	90.5%			
Malformation	1	0.8%		1	0.8%		1.31 (0.32-5.28)	0.14
Pre-term delivery	19	16%		19	9.9%			0.16

LBW, low birth weight; f_i_ = simple absolute frequency; RR = Relative risk; (Confidence level -CI = 95%).

### Pregnant women with malaria

Among the 118 pregnant women with vivax malaria, 109 (92.4%) initiated treatment on the day of symptom onset. The other nine initiated treatment up to five days after symptom onset. All initiated treatment on the same day of diagnosis of vivax malaria by the thick blood film. Comparing the mean Hb levels with increased placental thickness, low birth weight or pre-term births there were no significant difference between the groups of pregnant women with lower Hb levels and increased placental thickness (Table 5).

**Table 5:** **Comparison of haemoglobin levels relative to the ultrasound findings throughout the pregnancy**

	**Haemoglobin (mg/dL)**					
**Variables**	**<10.9**		**≥10.9**			
	**f**_**i**_	**%**	**f**_**i**_	**%**	**Total**	**p**
**Placental thickness (n = 118)**						
Yes	5	50	5	50	10	**0,381**
No	47	43.5	61	56.5	108	
**USG (n = 118)**						0.117
Abnormal findings	23	42.2	37	57.8	60	
Normal	29	50	29	50	58	
**LBW (n = 118)**						0.744
Yes	3	42.8	4	57.2	7	
No	49	44.2	62	55.8	111	
**PTL (n = 118)**						0.883
Yes	8	44.5	10	55.5	18	
No	44	44.0	56	56.0	100	

USG, ultrasonography; LBW, low birth weight, PTL, pre-term labour.

f_i_ = simple absolute frequency.

The time to the first ultrasound examination ranged from 0–15 days after the diagnosis of the symptoms, with a mean of five days. Sixteen patients evolved with relapse throughout the follow-up period, however these patients did not present any ultrasound differences as compared to those that did not relapse.

## Discussion

The present study evaluates foetal and placental ultrasound changes in pregnant women with vivax malaria in Latin America. Significant differences in the placental thicknesses between the malaria and non-malaria groups were revealed via placental ultrasound. Ultrasound studies of placental thickness have reported that placentas with increased thicknesses are more likely to be associated with hydrops, molar pregnancy, other infections during pregnancy, triploidy, maternal diabetes mellitus, anaemia and aneuploidy [36,38].

Increased placental thickness does not comprise a specific diagnosis of any disease. However, it can act as an alert to identify foetuses at high risk of disease resulting in inflammation, oedema or compensatory hypertrophy [36].

Lee *et al*. [21-23] studying placental thickness in the second trimester found that placental position could lead to an increased placental thickness. Despite that placental thickness may vary with the implantation site, there were no case of fundal or lateral placenta in the increased placental thickness group that would influence placental thickness.

Godfrey *et al*. [39] in their study of the effect of maternal anaemia and iron deficiency on the ratio of foetal weight to placental weight, Agboola [40] studying the effect of type and duration of anaemia on placental weight and villous anaemia, Baptiste-Roberts *et al*. [41] studying maternal risk factors for abnormal placental growth, found that increased placental thickness could be result in foetal hypoxaemia consequent to anaemia stimulating growth to impaired oxygen transportation [39]. There was no significant correlation between the pregnant women with regard to anaemia and increased placental thickness, and patients with diseases such as diabetes or infections were excluded from the present study, strengthening the hypothesis that increased placental thickness resulted from inflammation or oedema.

When using three-dimensional ultrasound to study placental volumes in pregnant women with vivax and falciparum malaria in Thailand, Rijken *et al.* [42] found no significant differences between the placental volumes of the pregnant women with vivax malaria and those without malaria [42]. No studies have been conducted on measurements of placental thickness in pregnant women with malaria, and the existing studies on vivax malaria and placentas address histopathology and do not seem to demonstrate that *P. vivax* causes inflammation in the intervillous spaces [43,44].

In a study of the effects of placental *P. falciparum* and *P. vivax* infections on placental histopathology in low malaria transmission areas, McGready *et al.* [45] found histopathological changes in a small proportion of the placentas from *P. falciparum-*infected patients, especially those who were infected soon before delivery. The *P. vivax* infections in this study by McGready *et al.* [45] were associated only with the presence of a malarial pigment in some placentas, indicating that prompt and adequate parasitaemia treatment during gestation would limit placental disease, emphasizing the importance of treating the infection with anti-malarial drugs during gestation [45]. Studies that correlate the placental thickness on ultrasound with histopathology should be performed to define the cause of the increased placental thickness observed in the present study.

The foetal biometry measurements, including BPD, FL, AC, and foetal weight, are important tools with which to evaluate gestational age, IUGR and detecting obstetrics problems. It is fundamental to know the correct gestational age measured by a first trimester ultrasonography and compare the foetal biometry and the weight expected for the gestational age, as was done in this study [46-48].

In the present study, most of the pregnant women developed malaria during the second and third trimesters of gestation, and an asymmetric growth restriction with an abdominal circumference reduction was expected, but not observed. In both groups, the weights were similar to those considered adequate in the 50^th^ percentile for the 38- to 40-week age range, according to a study by Pedreira *et al*. on gestational age-based foetal weight parameters in Brazil [49].

Some studies showed that recently infected women supposedly have a tendency towards more favourable perinatal outcomes because of the absence of immunological memory [7,50,51]. In the present study, most of the patients were recently infected, and there was no association between recent infection and ultrasound or neonatal changes. Given their existing lower physiological immunity, non-immune pregnant women would supposedly have a tendency towards a higher susceptibility to malaria and more severe conditions with recent infections, which was not observed [6,51].

In a study of foetal biometry via umbilical artery and uterine artery Doppler in women with vivax malaria in Msambweni, Dent *et al.* concluded that earlier treatment yielded fewer or even no effects throughout the gestation [52]. Rikjen, studying ultrasound evidence of early foetal growth restriction after maternal malaria infection, found that intermittent preventive treatments are the main strategy to prevent malaria, reduce anaemia and low birth weight [53]. Schmiegelow, studying malaria and foetal growth alterations in the third trimester of pregnancy, concluded that the effects on foetal growth often occurred after a considerable delay in women who were diagnosed with malaria at the first antenatal visit and who subsequently strictly followed the antenatal programme and the recommended measures to prevent malaria infections [54]. Thus, active malaria screening must be encouraged in pregnant women to ensure the early implementation of treatment and obtain a low morbidity rate and good perinatal outcomes.

The antenatal supplementation with daily iron is effective in reducing the risk of low birth weight [55,56]. This could also explain the lack of differences between the two groups at the end of follow-up, as evidence of the benefit of this supplementation, however this finding needs further study in endemic areas, such as those in Latin America. Another possible determinant of the lack of major differences between the two groups was that women initiated treatment very early, due to their easy access to the health system in Manaus and surrounding areas. This probably reinforces the impact of early diagnosis in endemic areas to this population.

It is worth noting that no changes in the demographic data were observed among the groups of pregnant women, suggesting that the groups were homogeneous and that epidemiological factors were not confounding factors in this study.

In the present study, the changes in the placental thickness, although significant, did not cause foetal repercussions at birth. There were no morphological or foetal changes that could be considered characteristic of foetal involvement in vivax malaria. The fact that the ultrasound examinations agreed with those from a cohort group of unexposed pregnant women was most likely due to the area being of low endemicity, and therefore, all pregnant women in the present study participated in follow-ups in an active detection system that allowed early diagnosis and treatment of new malarial episodes. The treatment and control of infections and relapses ensured that the behaviour of the infected group was similar to that of the group of pregnant women without malaria. In these study, malaria was an acute and non-chronic event with acute consequences that, in most cases, did not have any consequences and retreated spontaneously after treatment, thus demonstrating the benefits of adequate treatment and close follow-up of infected cases. All women were treated with chloroquine, and recent data showed that *P. vivax* chloroquine-resistance is supposed to be around 5% [57].

Limitations of the present study were low power of the study in order to estimate the frequency of low birth weight in *P. vivax* infection; only thick blood smears were performed; PCR to diagnose low parasitaemia could not be performed; patients performed weekly prophylaxis with chloroquine without supervision; some infections such as syphilis, toxoplasmosis and rubella were excluded because are routinely tested in antenatal care, but other infections, although rare, were not excluded and could have lead to increased placental thickness; the examinations were performed by an ultrasound examiner who was not blinded to the presence or absence of infection, leading to bias. Furthermore, variables derived from ultrasound biometry followed the Hadlock reference table from a North American population, impairing a precise estimation in the Amazonian population. However, using a control group this limitation was surpassed.

## Conclusions

The placental thickness changes were significant but caused no foetal repercussions at birth. The ultrasound findings except placental thickness were similar in both groups, possibly because this is a low-endemic area and the pregnant women in the study were followed up in an active detection system that allowed early diagnosis and treatment of new malaria episodes.

From the public health perspective, the finding of increased placental thickness could be a sentinel marker of malarial infection and this information could be useful for those who perform ultrasound examinations in remote areas, alerting the need of thick blood smear systematically. However, further studies in other areas are needed to confirm this finding.

## Additional files

Additional file 1:
**Characteristics of the pregnant women with placental thickness.**
Additional file 2:
**Comparison of the study groups with regard to the Z scores of the BPD, HC, AC, and FL of the pregnant women according to the trimester of recruitment.**
