# Supplementary material for: Ultrasound findings in pregnant women with uncomplicated vivax malaria in the Brazilian Amazon: a cohort study
Source: Malar J. 2015 Apr 8;14:144. doi: 10.1186/s12936-015-0627-1 (PMC4393585; doi:10.1186/s12936-015-0627-1)
Supplement: Additional file 1: — Characteristics of the pregnant women with placental thickness. [file 12936_2015_627_MOESM1_ESM.doc]

**Additional file Table S1:** Characteristics of the pregnant women with placental thickness

| Case | Age  (years) | Parity | 1st malarial infection | Placental Thickness 1st US | **placental thickness** Other USG | Hb level | Weigh gain Kg | IUGR | GA labor weeks | Newborn weight |
| --- | --- | --- | --- | --- | --- | --- | --- | --- | --- | --- |
| **1** | 17 | 1/0 | Y | Y | Y | 10.6 | 11.3 | N | 41 | 3070g |
| **2** | 20 | 1/0 | N | Y | Y | 10.5 | 12.3 | N | 39 | 3650g |
| **3** | 30 | 7/6 | N | Y | Y | 10.0 | 11.9 | N | 40 | 3630g |
| **4** | 16 | 2/1 | Y | Y | Y | 11.2 | 12.6 | N | 40 | 3150g |
| **5** | 23 | 1/0 | Y | Y | Y | 10.3 | 14.5 | N | 38 | 4000g |
| **6** | 31 | 1/0 | N | Y | Y | 11.5 | 12.8 | N | 38 | 3510g |
| **7** | 25 | 6/5 | Y | Y | Y | 11.0 | 13.3 | N | 39 | 3848g |
| **8** | 18 | 2/0 | Y | Y | Y | 11.6 | 12.4 | N | 42 | 3450g |
| **9** | 17 | 2/1 | N | Y | Y | 10.4 | 10.9 | N | 37 | 3150g |
| **10** | 21 | 2/0 | Y | Y | Y | 10.0 | 10.1 | N | 34 | 2370g |

GA, gestational age; IGUR, intrauterine growth restriction; USG, ultrasonography; g, grams; Hb, hemoglobin; Y, yes; N, no
