# Supplementary material for: Ultrasound findings in pregnant women with uncomplicated vivax malaria in the Brazilian Amazon: a cohort study
Source: Malar J. 2015 Apr 8;14:144. doi: 10.1186/s12936-015-0627-1 (PMC4393585; doi:10.1186/s12936-015-0627-1)
Supplement: Additional file 2: — Comparison of the study groups with regard to the Z scores of the BPD, HC, AC, and FL of the pregnant women according to the trimester of recruitment. [file 12936_2015_627_MOESM2_ESM.doc]

**Additional file Table S2:** Comparison of the study groups with regard to the Z scores of the BPD, HC, AC and FL of the pregnant women according to the trimester of recruitment.

| **1ST USG after Malaria** | | | | | | | **Last USG before birth** | | | | | | | |
| --- | --- | --- | --- | --- | --- | --- | --- | --- | --- | --- | --- | --- | --- | --- |
| **Z score** | **Vivax** | | **Control** | |  |  | **Vivax** | | | **Control** | | |  |  |
| **1^st^ Trim** | f_i_ | % | f_i_ | % | Total | p | f_i_ | % | f_i_ | | % | Total | | p |
| BPD |  |  |  |  |  | 0.96* |  |  |  | |  |  | | 0.75* |
| <2dp | 1 | 20.0 | 4 | 80.0 | 5 |  | 3 | 30.0 | 7 | | 70.0 | 10 | |  |
| Normal | 12 | 32.4 | 25 | 67.6 | 37 |  | 10 | 31.3 | 22 | | 68.7 | 32 | |  |
| **HC** |  |  |  |  |  | 0.84* |  |  |  | |  |  | | 0,64* |
| <2dp | 1 | 16.7 | 5 | 83.3 | 6 |  | 1 | 14.3 | 6 | | 85.7 | 7 | |  |
| Normal | 12 | 33.3 | 24 | 66.7 | 36 |  | 12 | 34.3 | 23 | | 65.7 | 35 | |  |
| **AC** |  |  |  |  |  | 0.64* |  |  |  | |  |  | | 0.36* |
| <2dp | 1 | 14.3 | 6 | 85.7 | 7 |  | 1 | 11.1 | 8 | | 88.9 | 9 | |  |
| Normal | 12 | 34.3 | 23 | 65.7 | 35 |  | 12 | 36.7 | 21 | | 66.3 | 33 | |  |
| **FL** |  |  |  |  |  | 0.18* |  |  |  | |  |  | | 0,36* |
| <2dp | 1 | 9.1 | 10 | 90.9 | 11 |  | 2 | 15.4 | 11 | | 84.6 | 13 | |  |
| Normal | 12 | 38.7 | 19 | 61.3 | 31 |  | 11 | 38.0 | 18 | | 62.0 | 29 | |  |
| **2^nd^ Trim** |  | |  | |  |  |  |  |  | |  |  | |  |
| **BPD** |  |  |  |  |  | *0.28** |  |  |  | |  |  | | *0,19** |
| <2dp | 4 | 23.5 | 13 | 76.5 | 17 |  | 7 | 26.0 | 20 | | 74.0 | 27 | |  |
| Normal | 69 | 41.1 | 99 | 58.9 | 168 |  | 66 | 41.8 | 92 | | 58.2 | 158 | |  |
| **HC** |  |  |  |  |  | *0.97** |  |  |  | |  |  | | *0,75** |
| <2dp | 4 | 44.4 | 5 | 55.6 | 9 |  | 4 | 50.0 | 4 | | 50.0 | 8 | |  |
| Normal | 69 | 39.2 | 107 | 60.8 | 176 |  | 69 | 39.00 | 108 | | 61.0 | 177 | |  |
| **AC** |  |  |  |  |  | 0.82* |  |  |  | |  |  | | 0,79* |
| <2dp | 3 | 30.0 | 7 | 70.0 | 10 |  | 5 | 38.4 | 8 | | 61.6 | 13 | |  |
| Normal | 70 | 40.0 | 105 | 60.0 | 175 |  | 68 | 39.5 | 104 | | 60.5 | 172 | |  |
| **FL** |  |  |  |  |  | 0.50* |  |  |  | |  |  | | 0. 38* |
| <2dp | 4 | 26.7 | 9 | 73.3 | 13 |  | 2 | 20.0 | 8 | | 80.0 | 10 | |  |
| Normal | 69 | 28.9 | 103 | 71.1 | 172 |  | 71 | 40.6 | 104 | | 59.4 | 175 | |  |
| **3^rd^ Trim** |  |  |  |  |  |  |  |  |  | |  |  | |  |
| **BPD** |  |  |  |  |  | 0.65* |  |  |  | |  |  | | - |
| <2dp | 2 | 66.7 | 1 | 33.3 | 3 |  | 2 | 100 | - | | - | 2 | |  |
| Normal | 30 | 38.0 | 49 | 62.09 | 79 |  | 30 | 37.5 | 50 | | 62.5 | 80 | |  |
| **HC** |  |  |  |  |  | - |  |  |  | |  |  | | - |
| <2dp | - | - | - | - | - |  | - | - | - | | - | - | |  |
| Normal | 32 | 39.0 | 50 | 61.0 | 82 |  | 32 | 39.0 | 50 | | 61.0 | 82 | |  |
| **AC** |  |  |  |  |  | - |  |  |  | |  |  | | - |
| <2dp | - | - | - | - | - |  | - | - | - | | - | - | |  |
| Normal | 32 | 39.0 | 50 | 61.0 | 82 |  | 32 | 39.0 | 50 | | 61.0 | 82 | |  |
| **FL** |  |  |  |  |  | - |  |  |  | |  |  | | - |
| <2dp | - | - | 1 | 100.0 | 1 |  | - | - | - | | - | - | |  |
| Normal | 32 | 39.5 | 49 | 60.5 | 81 |  | 32 | 39.0 | 50 | | 61.0 | 82 | |  |

f_i_ = simple absolute frequency; * chi-square with *Yates* correction.

A bold, italicized p-value indicates a statistical difference at a 5% significance level.

+ Number of total ultrasound exams performed during each trimester. Trim. trimester **.**
